# In Italy, *healthy life expectancy* drop dramatically: from 2004 to 2008 there was a 10 years drop among newborn girls

**DOI:** 10.1186/1824-7288-38-19

**Published:** 2012-05-18

**Authors:** Valerio Gennaro, Giovanni Ghirga, Laura Corradi

**Affiliations:** 1 Descriptive Epidemiology and Cancer Registries, Epidemiology and Prevention Dept, National Cancer Research Institute, Largo Rosanna Benzi, 10 - 16132, Genova, Italy; 2 San Paolo General Hospital, Largo Donatori del sangue, 00053, Civitavecchia, Italy; 3 University of California, Primary Prevention Expert, Researcher Professor in Sociology of Health & Environment, Università della Calabria, Campus di Arcavacata - via Pietro Bucci, 87036, Arcavacata di Rende, Cosenza, Italy

## Abstract

**Introduction:**

In this short essay, we would like to address a severe divergence observed in Italy between Life Expectancy (LE) and Healthy Life Expectancy (Healthy LE) and a unique trend of worsening in Healthy LE, compared to the other European countries. Both issues emerge in recent data by EUROSTAT Report.

**Methods:**

The analysis used by the authors of the EUROSTAT report is based on Sullivan method which combines 2 type of variables: mortality and morbidity data.

**Results:**

While several European countries started to deal with comparable data about LE since 1960, in Italy, analogous data were available for the first time in EUROSTAT Report only in 1985. In Italy, in the period 1985-2008, there was a good progressive increase in L.E., following the best European values. Nevertheless, while until 2004 Italy was among the European best countries in terms of both LE and Healthy LE at birth, four years later in 2008 there was a shocking loss of 10 years of Healthy LE at birth in newborn girls. In the process, they lost their 2-years previous advantage with respect to males (the latter lost only 6 years of Healthy LE, in the same time span). Looking at healthy LE at age 65 in respect to 2004, Italian women in 2008 could expect to live healthy only about 7 years (as much as men) versus the almost 15 years of the European best values (14 years for men).

**Conclusions:**

It is legitimate to wonder why no one official comment has been produced as a reaction after the first year of spectacular decline in Healthy Life Years in Italy: in counter-tendency with European values, from 2004 to 2008 there is a clear evidence of a 10 years drop in Healthy LE among newborn girls. The problem has not been taken into consideration even when the situation clearly appeared to worsen in the following years, dropping 4-6 more years for males and females in 2006 (for newborn babies); two more years of healthy life expectancy have been lost between 2006 and 2007 for each gender. One more year of Healthy Life Expectancy is lost in 2008. And data have not been made available any more, since then, from Italy.

## Dear editor

**Figure 1:**
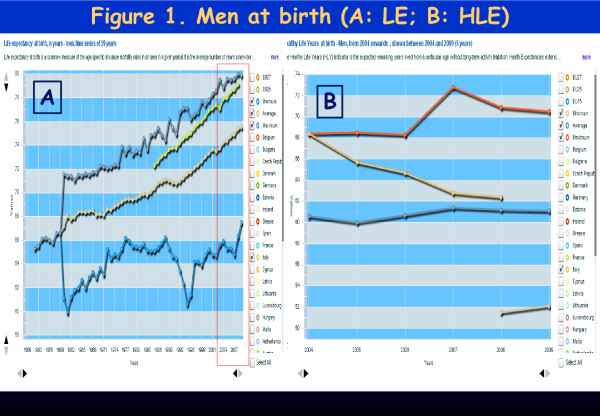
**Life Expectancy (LE) and Healthy Life Expectancy (HLE) at birth (in years) - Women 2004-2008 (5 years).** Figure A: LE for European (minimum, maximum, average) and Italian women (green line). Figure B: HLE for European (minimum, maximum, average) and Italian women (orange line in declining trend).

**Figure 2:**
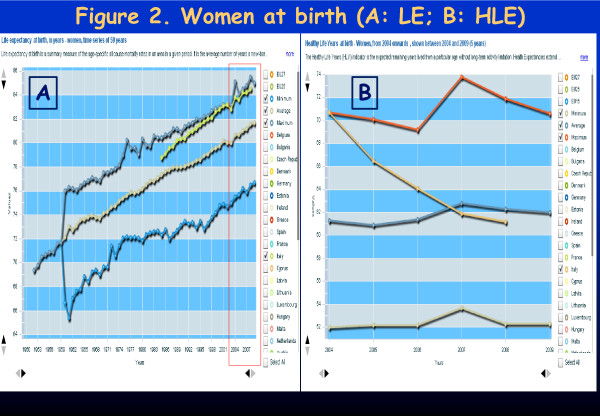
**Life Expectancy (LE) and Healthy Life Expectancy (HLE) at birth (in years) - Women 2004-2008 (5 years).** Figure A: LE for European (minimum, maximum, average) and Italian women (green line). Figure B: HLE for European (minimum, maximum, average) and Italian women (orange line in declining trend).

We would like to address a severe divergence observed in Italy between Life Expectancy (LE) and Healthy Life Expectancy (Healthy LE); and a unique trend of worsening in Healthy LE, compared to the other European countries. Both issues emerge in recent data by EUROSTAT Report [1] (Figures 1 and 2).

While LE represents the mean number of years still to be lived by a person (at birth and at age 65), subjected throughout the rest of his/her life to the current mortality conditions (age-specific probabilities of dying), Healthy LE is measured through the number of years a person is expecting to live in healthy conditions (at birth and at age 65) i.e., without severe illnesses, a disability free life [2].

While several European countries started to deal with comparable data about *Healthy* LE since 1960, in Italy, analogous data were available for the first time in EUROSTAT Report only in 1985. Until now (26 years late) in most popular scientific arenas, Italians have been made to believe life length is the most desirable output: no information nor debate dare to address the differences between *LE* and *Healthy LE.* Consequently*,* up to the present, the public opinion still perceives a *long* life as equivalent of a *good* life.

In Italy, in the period 1985–2008 (23 years), there was a good progressive increase in L.E., following the best European values.

Until 2004 Italy was among the European best countries in terms of *Healthy LE* at birth – women were displaying 2 years of advantage with respect to men (about 70 *versus* 68 years). Four years later, in 2008, we assist to the **shocking loss of 10 years of*Healthy LE*at birth in newborn girls**. In the process, they lose their 2-years previous advantage with respect to males (**the latter lost*only*6 years of Healthy LE,** in the same time span). Females fall one year below males value and below the females European average (corresponding to about 62 years). In other words, in 2004 any newborn girl could expect to live healthy up to about 70 and a boy up to 68 years. After only 4 years, in 2008 newborn girls could expect to live healthy only up to 61 and a boy up to age 62. Males find themselves 1 year above the average European standard (61 years).

The gap between *LE* and *Healthy LE* in Italy is a critical issue. In synthesis, while large emphasis has been shown in Italy for the 3-month-a-year increasing in LE, no information has been given about Healthy LE, an important indicator*,* which shows a decline among newborn: 18 months for males and 27 months for females during 4 years time span (2004–2008)

The problem has not been taken into consideration by the Italian Government even when the situation clearly appeared to worsen in the following years, dropping 4–6 more years for males and females in 2006 (for newborn babies); two more years of healthy life expectancy have been lost between 2006 and 2007 for each gender. One more year of Healthy Life Expectancy is lost in 2008. At this writing, **data have not been made available any more, since then, from Italy.**

Any loss in health has important second order effects. These will include an altered pattern of resource allocation within the health-care system, as well as wider ranging effects on consumption and production throughout the economy. It is important for policy-makers to be aware of the cost of doing too little to prevent ill-health, resulting in the use of limited health resources for the diagnosis, treatment, and management of preventable illness and injuries. Due to high annual cost to the National Health Service, in Italy more than 100 bln euro (Ministero della Salute – Sistema Informatico Sanitario), improvement in health would save a large amount of money. A very important fact during this severe economic crisis.

As concerned scientists, we believe it is an urgent issue to address the **Italian case in the international debate**. We are committed to operate against prejudice and misconceptions around quantity and quality of life; and we are dedicated in increasing the awareness around the dramatic drop in the Italian Healthy Life Years – both in the scientific community, and in society at large.
